# Epstein-Barr virus infection is associated with a higher Child-Pugh score and may predict poor prognoses for patients with liver cirrhosis

**DOI:** 10.1186/s12876-019-1021-1

**Published:** 2019-06-18

**Authors:** Jianhua Hu, Xiaoli Zhang, Guodong Yu, Huan Cai, Jueqing Gu, Menglin Hu, Dairong Xiang, Jiangshan Lian, Liang Yu, Hongyu Jia, Yimin Zhang, Yida Yang

**Affiliations:** State Key Laboratory for Diagnosis and Treatment of Infectious Diseases, Collaborative Innovation Center for Diagnosis and Treatment of Infectious Diseases, The First Affiliated Hospital, College of Medicine, Zhejiang University, 79 QingChun Road, Hangzhou, Zhejiang China

**Keywords:** Epstein-Barr virus, Infection, Liver cirrhosis, Risk factors, Child-Pugh score, Liver function

## Abstract

**Background:**

Studies on Epstein-Barr virus (EBV) have focused mostly on neoplastic disease. Few studies have considered immunocompetent patients who are not severely immunocompromised. Liver cirrhosis is associated with various levels of immune dysfunction. In the current study, we determined EBV infection rates, the influence on liver function, and analyzed the risk factors for death in patients with liver cirrhosis.

**Methods:**

The medical records of patients diagnosed with liver cirrhosis between 1 January 2014 and 31 December 2016 were reviewed. Patients who were or were not infected with EBV were enrolled in this study. Liver functions were compared. The risk factors for 28-, 90-, and 180-day mortality rates were analyzed by univariate and multivariate logistic regression.

**Results:**

The medical records hospitalized patients diagnosed with liver cirrhosis were reviewed. Of these patients, 97 had assessed EBV deoxyribonucleic acid (DNA) and 36 (37.1%) patients were EBV DNA-positive. The age of the EBV-infected patients was older than patients not infected with EBV. EBV-infected patients had a lower level of albumin, and a lower albumin-to-globulin ratio (*P* = 0.019 and *P* = 0.013, respectively). EBV-infected patients had higher Child-Pugh scores (*P* = 0.033) and higher acute-on-chronic liver failure (ACLF) rate (*P* = 0.050). The Child-Pugh score and ACLF were the risk factors for the 28-, 90-, and 180-day mortality rates.

**Conclusions:**

This study revealed that patients with liver cirrhosis had higher EBV infection rates, especially patients > 60 years of age, which likely reflected viral reactivation. And liver injury was aggravated in EBV-infected patients. Thus, EBV infection indirectly influenced the prognosis of EBV-infected patients by increasing the Child-Pugh score and ACLF rate.

## Background

Epstein-Barr virus (EBV), a γ-human herpesvirus, is one of the most common pathogens, with seropositivity rates approaching 90% [1, 2]. Primary EBV infections primarily occur in childhood, manifesting as infectious mononucleosis with fever, angina, lymphadenectasis, hepatomegaly, and splenomegaly. After primary infections, EBV may latent in B cells [3]. Reduplication of these latent viruses occur when immune function decreased or impaired.

Studies on EBV have focused on patients with neoplastic disease [4] and post-transplant lymphoproliferative disorders after hematopoietic stem cell transplantation [5–7]. A limited number of studies have involved immunocompetent patients who were not severely immunocompromised [8, 9].

EBV is a non-eosinophilic virus, but it is also a pathogen that causes liver injury, and even liver failure [8–10]. Autoimmune liver diseases, including autoimmune hepatitis, primary biliary cirrhosis, and primary sclerosing cholangitis, have a potential causative link with EBV [11]. Vine et al. [12] reported that in patients presenting with jaundice/hepatitis, EBV hepatitis is an uncommon diagnosis and causes a self-limiting hepatitis. The diagnosis is suggested by the presence of a lymphocytosis and/or splenomegaly. The majority of patients do not have infectious mononucleosis. Ulug et al. [13] have reported a case of acute hepatitis associated with acute EBV infection. Also, Gupta et al. [14] have described two cases of acute hepatitis after EBV infection.

Liver cirrhosis is the final stage of chronic liver disease from any cause, and is associated with immune dysfunction, which is referred to as cirrhosis-associated immune dysfunction syndrome [15]; however, the primary infection or an EBV infection is unknown in the case of mild immune dysfunction that occurs in patients with liver cirrhosis.

Ascites, spontaneous bacterial peritonitis (SBP), hepatic encephalopathy, gastrointestinal bleeding, hepatocellular carcinoma, and hepatorenal syndrome are the main complications of cirrhosis and the most common cause of death [16–22]. It is well-known that the Child-Pugh score [16, 23] and model for end-stage liver disease (MELD) score [16, 24] can be used to accurately assess liver function and prognoses of patients with liver cirrhosis. Other studies [22, 25–27] have reported that hepatocellular carcinoma, hypoalbuminemia, bacterial infections, and secondary infections may predict a worse prognosis in liver cirrhosis; however, the above studies were based on univariate analyses. In addition, apart from the above risk factors, it is unclear whether or not EBV infection influences prognosis in patients with liver cirrhosis. In this study, we aim to retrospective analyze the EBV infection rate and the influence on liver function, and also analyze the risk factors for mortality in patients with liver cirrhosis.

## Methods

### Patients

This study was performed in a retrospective manner. The databases of patients with liver cirrhosis admitted to the First Affiliated Hospital (College of Medicine, Zhejiang University) between 1 January 2014 and 31 December 2016 were reviewed. Patients who were or were not infected with EBV were enrolled in this study. The demographic, clinical characteristics, and some experimental results (liver function, coagulation function, and EBV deoxyribonucleic acid (DNA) level) and ultrasonic scanning results (mainly included liver size and spleen size) during hospitalization were reviewed by a trained team of physicians and entered into a computerized system in duplicate. We also collected patient mortality data (28-, 90-, and 180-day transplant-free mortality) by reviewing medical documents or telephone follow-ups. Mortality was assessed during the hospital stay and after hospital discharge.

### Enrollment criteria

Patients who were diagnosed with liver cirrhosis and had EBV DNA assessed were selected.

The diagnosis of cirrhosis was based on the following, as previously described [27, 28]: liver biopsy; clinical evidence of decompensation or varices; radiological evidence of liver nodularity; and intra-abdominal variations in a patient with chronic liver disease.

### Exclusion criteria

Patients with the following criteria were excluded: (1) patients in whom the level of EBV DNA was not determined; (2) patients < 18 years of age; (3) patients with acquired immune deficiency syndrome (AIDS); (4) patients with other types of tumors besides hepatocellular carcinoma; (5) a history of liver transplantation; (6) patients who require long-time corticosteroids treatments (prolonged use of corticosteroids at a mean minimum dose of 0.3 mg/kg/day of prednisone equivalent for > 3 weeks) [29]; (7) patients who require immunosuppressive therapy (mainly including recognized T cell immunosuppressants, such as cyclosporine, TNF-*α*blockers, specific monoclonal antibodies) [29].

### Definition

Bacterial infections were defined as follows [27, 28]: (1) pneumonia was defined as a new pulmonary infiltrate with fever, respiratory symptoms (cough, sputum production, dyspnea, and pleuritic pain), findings on auscultation (rales or crepitation), and/or a leukocyte count > 10,000/mm3 or < 4000/mm3; (2) spontaneous bacterial peritonitis was defined as ascitic fluid polymorphonuclear cells > 250/μL with/without a positive fluid culture; (3) urinary tract infections were defined as urine white blood cell (WBC) > 15/high power field with positive culture and symptoms of urinary irritation; (4) skin and soft tissue infections were defined as a fever and cellulitis associated with leukocytosis; and (5) undetermined infections were defined as bacterial infections, but without positive cultures or evidence of organ involvement.

EBV infection was referred to as EBV DNA detected in whole blood samples by a quantitative polymerase chain reaction assay.

Liver size referred to the measurement of the inferior oblique diameter of rib margin of right liver. Spleen size referred to the measurement of the thickness of spleen. Within liver was referred as inferior oblique diameter of rib margin of right liver<10.0 cm and splenomegaly was referred as the thickness of spleen > 4.0 cm, which were measured by ultrasonic scanning.

### Quantitative polymerase chain reaction assay for EBV DNA

According to the manufacturer’s instructions, EBV DNA was extracted from blood samples using DNA extraction kit (Daan Gene Co.Ltd., Zhongshan University, China). Quantitative polymerase chain reaction (PCR) was detected on a real-time PCR system (Stratagene MX3000P; Agilent Technologies, Santa Clara, CA, USA), just as our previous reported [30].

### Liver function, child-Pugh score, model for end-stage liver disease (MELD) score and acute-on-chronic liver failure (ACLF)

Liver function, including albumin, the albumin-to-globulin ratio (A/G), alanine aminotransferase (ALT), aspartate aminotransferase (AST), total bilirubin (TB), direct bilirubin (DB), alkaline phosphatase (ALP), γ-glutamyl transpeptidase (GGT), and cholinesterase (CHE), was reviewed.

The Child-Pugh score [23] and MELD score [24] are used to accurately assess liver function and prognosis of patients with liver cirrhosis. We calculated the Child-Pugh and MELD scores of the patients according to the collected data.

Diagnostic criteria and grading of ACLF based on the chronic liver failure-sequential organ failure assessment (CLIF-SOFA score) [31]. And CLIF-C Score [32] was also assessed.

### Risk factors associated with 28-, 90-, and 180-day transplant-free mortalities in patients with EBV-IgG positive liver cirrhosis

In this study, the 28-, 90-, and 180-day mortality rates were determined from the first day of admission to the hospital to 28, 90, and 180 days.

According to previous studies on mortality risk factors for patients with liver cirrhosis [16–22, 25, 26], we analyzed the 28-, 90-, and 180-day mortality rates by univariate and multivariate logistic regression, including the Child-Pugh score, MELD score, ACLF, age, ascites, hepatic encephalopathy, gastrointestinal bleeding, hepatocellular carcinoma, hepatorenal syndrome, bacterial infection, hypoalbuminemia, EBV infection, international normalized ratio (INR), and serum creatinine (Scr).

### Statistical analysis

Statistical analyses were performed using SPSS software. The results are expressed as the mean ± standard deviation (SD), and percentage. The means for continuous variables were compared using an independent group Student’s t-test, for which the data were normally distributed; otherwise, the Mann-Whitney U test was used. Categorical variables were analyzed by the chi-square test or the Fisher’s exact test. The risk factors for mortality were analyzed by univariate and multivariate logistic binary regression. All *p*-values were based on a two-tailed test of significance. Statistical significance was always defined as a p-value < 0.05.

## Results

### Demographic, clinical characteristics, and EBV infection

**Fig. 1:**
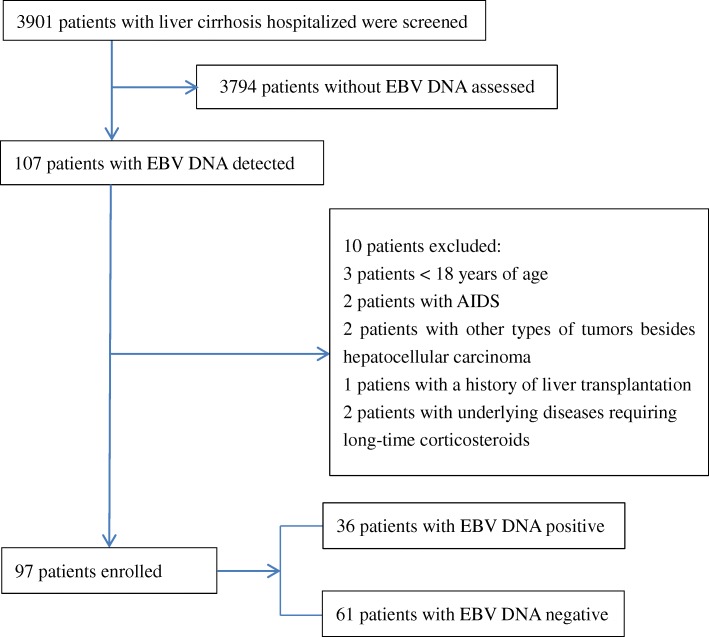
Flow chart of patient selection in this study

We identified 3901 hospitalized patients who were diagnosed with liver cirrhosis from 1 January 2014 to 31 December 2016. 3794 patients were excluded because EBVDNA was not assessed, and 10 patients were exclued because of various reasons (Fig. 1). A total of 97 patients with EBVDNA assessed were enrolled (Fig. 1), of which 36 (37.1%) were EBV DNA-positive, with a mean of 9573.4 copies/mL. The patients consisted of 52 males and 45 females who had a mean age of 55.9 years; 43.3% of the patients were at least 60 years of age. Hepatitis B infection caused cirrhosis in 51.6% of the patients, 68% of patients had ascites, and 26.8% of patients had bacterial infections. 13.4% patients had ACLF, of which 3 patients (3.1%) were in grade 1, 9 patients (9.3%) were in grade 2, 1 patients (1.0%) were in grade 3. The livers were reduced and the spleens were enlarged in all patients in this study. The mean size of liver was 8.52 ± 0.791 cm, and the mean size of spleen was 4.87 ± 0.815; There was no significant difference in liver and spleen size between EBV infected and uninfected patients. The demographic and clinical characteristics of the participants are presented in Table 1.

**Table 1:** Characteristics of patients with cirrhosis

Variable	Value
Patients	97
Sex (M/F)	52/45 (53.6%/46.4%)
Age (mean ± standard deviation, years)	55.9 ± 12.32
<60 yr	55(56.7%)
≥ 60 yr	42(43.3%)
Cause of liver cirrhosis	
Hepatitis B virus infection	50(51.6%)
Hepatitis C virus infection	1(1.0%)
Alcoholism	10(10.3%)
Primary biliary cirrhosis	12(12.4%)
Autoimmune hepatitis	6(6.2%)
Wilson’s Disease	1(1.0%)
Hepatolith	1(1.0%)
Schistosomiasis Cirrhosis	2(2.1%)
Cryptogenic Cirrhosis	14(14.4%)
Underling Conditions	
Hypertension	18(18.6%)
Diabetes Mellitus	18(18.6%)
Coronary Atherosclerotic Heart Disease	3(3.1%)
Chronic Bronchitis and Emphysema Pulmonum	2(2.1%)
Chronic kidney disease	2(2.1%)
Complications of liver cirrhosis	
Ascites	66 (68.0%)
Hepatic encephalopathy	21 (21.6%)
Hepatocellular carcinoma	7 (7.2%)
Hepatorenal syndrome	7 (7.2%)
Gastrointestinal bleeding	8 (8.2%)
Bacterial infection	30 (30.9%)
Pneumonia	22 (22.7%)
SBP	1 (1.0%)
Pneumonia and SBP	2 (2.1%)
Urinary tract infection	1 (1.0%)
Skin and soft tissue infection	1 (1.0%)
Undetermined infection	3 (3.1%)
ACLF	13 (13.4%)
ACLF grade 1	3 (3.1%)
ACLF grade 2	9 (9.3%)
ACLF grade 3	1(1.0%)
Transplant-free mortality	
28-days mortality	20 (20.6%)
90-days mortality	24 (24.7%)
180-days mortality	29 (29.9%)

*SBP* spontaneous bacterial peritonitis

### Liver function, child-Pugh score, MELD score and ACLF

All indices of liver function, including ALT, AST, TB, DB, CHE, ALP, and GGT, did not differ significantly (*P* > 0.05) between EBV-infected and -non-infected patients; however, the albumin level was lower in EBV-infected patients (30.92 ± weve1 g/L) than non-infected patients (33.65 ± 5.199 g/L); this difference was statistically significant (*P* = 0.019). Also, the difference in A/G was statistically significant (*P* = 0.013); specifically, EBV-infected patients had a lower A/G (1.05 ± 0.366) than non-infected patients (1.27 ± 0.432). For the EBV-infected and -non-infected patients, the Child-Pugh scores were 9.83 ± 2.864 and 8.64 ± 2.470, respectively; EBV-infected patients had higher Child-Pugh scores (*P* = 0.033). Nevertheless, the MELD scores of the two groups were also not significantly different (P > 0.05). (Table 2).

**Table 2:** Liver function, Child-Pugh score, and MELD score between EBV infected and un-infected patients

	EBV infected (*n* = 36)	EBV un-infected (*n* = 61)	P
Sex(male)	18 (50%)	34 (55.7%)	0.584
Age (years)	59.69 ± 12.540	53.69 ± 11.725	0.020
≥ 60 yr	22 (61.1%)	20 (32.8%)	0.007
Complications of liver cirrhosis			
Ascites	26 (72.2%)	40 (65.6%)	0.498
Hepatic encephalopathy	8 (22.2%)	13 (21.3%)	0.916
Hepatocellular carcinoma	2 (5.6%)	5 (8.2%)	> 0.99
Bacterial infection	15 (41.7%)	15 (24.6%)	0.079
Gastrointestinal bleeding	3 (8.3%)	5 (8.2%)	> 0.99
Hepatorenal syndrome	2 (5.6%)	5 (8.2%)	> 0.99
Transplant-free mortality			
28-days mortality	9 (25.0%)	11 (18.0%)	0.413
90-days mortality	10 (27.8%)	14 (23.0%)	0.595
180-days mortality	13 (36.1%)	16 (26.2%)	0.304
Liver function			
ALT (U/L)	103.00 ± 146.315	161.25 ± 279.798	0.182
AST (U/L)	110.75 ± 104.110	138.39 ± 174.436	0.331
Albumin (g/L)	30.92 ± 5.861	33.65 ± 5.199	0.019
A/G	1.05 ± 0.366	1.266 ± 0.432	0.013
TB (uoml/L)	173.94 ± 160.474	163.85 ± 172.967	0.776
DB (umol/L)	117.03 ± 111.236	110.97 ± 122.289	0.808
CHE(U/L)	2914.58 ± 1654.627	3638.16 ± 1859.968	0.057
ALP(U/L)	149.61 ± 141.881	135.52 ± 66.467	0.509
GGT (U/L)	111.19 ± 154.451	126.75 ± 164.061	0.646
Liver size	8.58 ± 0.826	8.49 ± 0.774	0.612
Spleen size	4.78 ± 1.369	4.47 ± 1.454	0.299
Child-pugh Score	9.83 ± 2.864	8.64 ± 2.470	0.033
Meld Score	18.33 ± 22.693	12.50 ± 9.995	0.084
ACLF	8 (22.2%)	5 (8.2%)	0.050
ACLF grade 1	2 (5.6%)	1 (1.6%)	0.553
ACLF grade 2	6 (16.7)	3 (4.9%)	0.073
ACLF grade 3	0 (0.0%)	1 (1.6%)	> 0.99

*ALT* alanine aminotransferase, *AST* aspartate aminotransferase, *A/G* Albumin/Globulin, *TB* total bilirubin, *DB* direct bilirubin, *GGT* γ-glutamyl transpeptadase, *CHE* cholinesterase, *ALP* alkaline phosphatase, *Meld score* model for end-stage liver disease score, *ACLF* acute-on-chronic liver failure

There were 8 patients had ACLF in patients with EBV infected, including 2 patients with grade 1 and 6 patients with grade 2. There were 5 patients had ACLF in patients with EBV un-infected, including 1 patients with grade 1, 3 patients with grade 2 and 1 patients with grade 3. patients with EBV infected had higher rate of ACLF. Compared to patients with EBV un-infected, patients with EBV infected had more higher ACLF rate (*P* = 0.050). However, there were no significant difference in ACLF grade between these two groups. (Table 2).

CLIF-C Score was also calculated for these 13 patients with ACLF, the mean score was 11.46 ± 1.050. And the mean score for EBV infected (8 patients) and un-infected patients (5 patients) were 11.50 ± 0.756 and 11.40 ± 1.517. there were no significant difference between the two groups (*P* = 0.896).

### Risk factors associated with 28-, 90-, and 180-day transplant-free mortalities in liver cirrhosis patients

In this study, we found that 20 (20.6%), 24 (24.7%), and 29 (29.9%) patients died within 28, 90, and 180 days, respectively.

Univariate and multivariate logistic regression were used to assess the factors studied and the possible relationships to mortality. For 28- and 90-day transplant-free mortality in univariate analysis, age, ascites, gastrointestinal bleeding, hepatocellular carcinoma, hepatorenal syndrome, bacterial infection, EBV infection, and serum creatinine (Scr) were shown not to be risk factors; however, the Child-Pugh score, MELD score, ACLF, hepatic encephalopathy, hypoalbuminemia, and INR were shown to be risk factors. Of note, we found that the Child-Pugh score and ACLF were the risk factors by multivariate logistic regression. Based on univariate analysis, the Child-Pugh score, MELD score, ACLF, hepatic encephalopathy, hypoalbuminemia, INR, ascites, and hepatocellular carcinoma were shown to be risk factors for 180-day transplant-free mortality; however, we also found that the Child-Pugh score and ACLF were the risk factors by multivariate logistic regression. (Tables 3, 4, 5).

**Table 3:** Univariate and multivariate logistic regression analysis of risk factor associated with 28-days transplant-free mortality

Variable	Univariate			Multivariate		
	OR	95% CI	P	OR	95% CI	P
Child-Pugh score	1.668	1.286–2.164	0.000	29.553	4.783–182.605	< 0.001
Meld score	1.057	1.003–1.115	0.038			
ACLF	45.833	8.735–240.500	< 0.001	1.499	1.126–1.995	0.006
Hypoalbuminemia	11.479	1.419–90.339	0.020			
Hepatic encephalopathy	3.282	1.120–9.617	0.030			
INR	7.249	2.257–23.282	0.001			

*ACLF* acute-on-chronic liver failure, *INR* international normalized ratio

**Table 4:** Univariate and multivariate logistic regression analysis of risk factor associated with 90-days transplant-free mortality

Variable	Univariate			Multivariate		
	OR	95% CI	P	OR	95% CI	P
Child-Pugh score	1.606	1.270–2.029	0.000	18.410	3.162–107.198	0.001
Meld score	1.052	1.002–1.104	0.040			
ACLF	30.038	5.953–151.560	< 0.001	1.460	1.140–1.871	0.003
Hypoalbuminemia	6.844	1.493–31.372	0.013			
Hepatic encephalopathy	5.331	1.877–15.142	0.002			
INR	6.705	2.179–20.638	0.001			

*ACLF* acute-on-chronic liver failure, *INR* international normalized ratio

**Table 5:** Univariate and multivariate logistic regression analysis of risk factor associated with 180-days transplant-free mortality

Variable	Univariate			Multivariate		
	OR	95% CI	P	OR	95% CI	P
Child-Pugh score	1.733	1.362–2.205	0.000	1.551	1.212–1.983	< 0.001
Meld score	1.052	1.005–1.101	0.030			
ACLF	20.167	4.095–99.312	< 0.001	14.631	2.375–90.132	0.004
Hypoalbuminemia	5.707	1.571–20.734	0.008			
Hepatic encephalopathy	4.627	1.671–12.819	0.003			
Ascites	4.116	1.288–13.154	0.017			
Hepatocellular carcinoma	6.875	1.250–37.825	0.027			
INR	8.271	2.587–26.447	0.000			

*ACLF* acute-on-chronic liver failure, *INR* international normalized ratio

## Discussion

EBV is an opportunistic infectious virus that is tumorigenic. Indeed, most research has focused on opportunistic infection after transplantation or neoplasia [4–7]; however, there are no reports involving EBV infections in patients with liver cirrhosis. EBV seropositive rates are as high as 90% [1, 2]. Herein we report that EBV infections in patients with liver cirrhosis reached 37.1%, which is slightly lower than the post-transplantation infection rate [5].

Primary infections with EBV usually occurs during the first few years of life and is often asymptomatic. It is reported that 75% of young adults have typical infectious mononucleosis after primary EBV infections [33, 34]. After primary infections, EBV may latent in B cells. Reduplication of these latent viruses occur when immune function decreased.

By reviewing the medical records and laboratory data, we found that 68 patients had EB viral capsid antigen (EB-VCA) IgG, all of whom were EB-VCA IgG-positive. Although 29 patients did not have EB-VCA IgG, 100% of the patients with detectable EB-VCA IgG were seropositive. Primary EBV infections mostly occur in childhood. Thus, in this study, it is likely that EBV infection was a reactivation rather than a primary infections. Here 36 patients were EBV reactivation with lower level of reduplication (9573.4 copies/mL mean). And there was no obvious clinical symptoms. And there was no difference in liver and spleen size between EBV infected and uninfected patients. Vine et al. [12] reported that EBV hepatitis is an uncommon diagnosis and causes a self-limiting hepatitis. The majority of patients do not have infectious mononucleosis.

The EBV-infected patients were older than the non-infected patients. A greater number of EBV-infected patients were > 60 years of age. Thus, age was a risk factor for EBV infection, especially in patients > 60 years of age. This is similar to previous reports [12]. Vine et al. [12] reported that compared with infectious mononucleosis, EBV hepatitis affects an older age group, with nearly half of patients being aged more than 60 years.

There were no differences with respect to complications of liver cirrhosis between EBV-infected and -non-infected patients, including ascites, hepatic encephalopathy, gastrointestinal bleeding, hepatorenal syndrome, infections, and hepatocellular carcinoma. The 28-, 90-, and 180-day transplant-free mortality rates also did not differ between EBV-infected and -non-infected patients. Thus, EBV infection may not influence the occurrence of complications in patients with liver cirrhosis and may not affect short-term mortality.

EBV is a non-eosinophilic virus, that is also a pathogen that causes liver injury, and even liver failure [8–10]. In this study, we found that patients with EBV infected had more higher ACLF rate (*P* = 0.050). However, there was no significant difference in ACLF grade and CLIF-C Score between these two groups. So, patients with EBV infected may had higher ACLF rate.

We compared the liver function between EBV-infected and -non-infected patients, and found that the level of albumin and the A/G were lower in EBV-infected patients, and in those patients the phenomena of enzyme bilirubin separation was more apparent. The Child-Pugh and MELD scores could better assess liver function. In this study, we found that EBV-infected patients had higher Child-Pugh scores. Thus, EBV infection may aggravate liver damage. Although EBV has not been clearly shown in hepatocytes, increased levels of EBV DNA encoding transcripts have been found in lymphocytes infiltrating the liver and inducing inflammation and chronic hepatitis [35].

In this study, we found that hepatic encephalopathy, hypoalbuminemia, the level of INR, the Child-Pugh score, the MELD score and ACLF were risk factors for 28- and 90-day mortality rates. Ascites, hepatocellular carcinoma, hepatic encephalopathy, hypoalbuminemia, the INR, Child-Pugh score, MELD score, and ACLF were risk factors for the 180-day mortality rate, which is in agreement with previous reports [16, 25]; however, when the above risk factors were analyzed by multivariate analysis, the Child-Pugh score and ACLF were the risk factors for the 28-, 90-, and 180-day mortality rates.

We did not show that EBV infection directly affected prognoses; however, EBV-infected patients had higher Child-Pugh scores and ACLF, and the Child-Pugh score and ACLF were the risk factors for short-time mortality. Thus, EBV infection might indirectly influence the short-term prognosis.

This work was limited by a number of factors. First, the study consisted of only 97 patients. Second, a slight bias was present in retrospective studies. Above two limited factors will result a certain statistical bias. Third, only 68 patients had detectable EB-VCA IgG. Although all of the detected patients were EB-VCA IgG-positive, it was still impossible to distinguish between primary infections and reactivation in all patients. Forth, There are few or very week direct evidence of EBV and prognosis of liver cirrhosis. Fifth, patients with EBV-infected in this study did not undergo liver biopsy. We could not analyze the histological changes of patients with EBV-infected. Sixth, we can not showing survival curves for the Child-Pugh score (and for other risk factors), because the exact time of death for many patients is unknown. We only know whether they died in 28 days, 90 days or 180 days. To address these drawbacks, we should expand our sample and perform a prospective study in the future.

## Conclusion

This study revealed that liver cirrhosis patients had higher EBV infection rates, especially patients > 60 years of age, which likely represented reactivation. EBV-infected patients might have had aggravation of liver damage. EBV infection indirectly influenced the prognoses by increasing the Child-Pugh score and ACLF rate.

## Abbreviations

A/G: the albumin-to-globulin ratio
ACLF: acute-on-chronic liver failure
AIDS: acquired immune deficiency syndrome
ALP: alkaline phosphatase
ALT: alanine aminotransferase
AST: aspartate aminotransferase
CHE: cholinesterase
DB: direct bilirubin
DNA: deoxyribonucleic acid
EBV: Epstein-Barr virus
EB-VCA: EB viral capsid antigen
GGT: γ-glutamyl transpeptidase
INR: international normalized ratio
MELD: model for end-stage liver disease
PCR: polymerase chain reaction
SBP: spontaneous bacterial peritonitis
Scr: serum creatinine
SD: standard deviation
TB: total bilirubin
WBC: white blood cell

## References

[1] Taylor GS, Long HM, Brooks JM, Rickinson AB, Hislop AD (2015). The immunology of Epstein-Barr virus-induced disease. Annu Rev Immunol.

[2] Maeda A, Sato T, Wakiguchi H (2006). Epidemiology of Epstein-Barr virus (EBV) infection and EBV-associated diseases. Nihon Rinsho.

[3] Vouloumanou EK, Rafailidis PI, Falagas ME (2012). Current diagnosis and management of infectious mononucleosis. Curr Opin Hematol.

[4] Ma J, Li JH, Hao YM, Nie YZ, Li ZS, Qian MR, Liang QY (2017). Differentiated tumor immune microenvironment of Epstein-Barr virus-associated and negative gastric cancer: implication in prognosis and immunotherapy. Oncotarget..

[5] Fan J, Jing M, Yang M, Xu L, Liang H, Huang Y, Yang R (2016). Herpesvirus infections in hematopoietic stem cell transplant recipients seropositive for human cytomegalovirus before transplantation. Int J Infect Dis.

[6] Peric Z, Cahu X, Chevallier P, Brissot E, Malard F, Guillaume T, Delaunay J (2011). Features of Epstein-Barr virus (EBV) reactivation after reduced intensity conditioning allogeneic hematopoietic stem cell transplantation. Leukemia..

[7] Kullberg-Lindh C, Mellgren K, Friman V, Fasth A, Ascher H, Nilsson S, Lindh M (2011). Opportunistic virus DNA levels after pediatric stem cell transplantation: serostatus matching, anti-thymocyte globulin, and total body irradiation are additive risk factors. Transpl Infect Dis.

[8] Mellinger JL, Rossaro L, Naugler WE, Nadig SN, Appelman H, Lee WM, Fontana RJ (2014). Epstein-Barr virus (EBV) related acute liver failure: a case series from the US acute liver failure study group. Dig Dis Sci.

[9] Zhang W, Chen B, Chen Y, Chamberland R, Fider-Whyte A, Craig J, Varma C (2016). Epstein-Barr virus-associated acute liver failure present in a 67-year-old immunocompetent female. Gastroenterology Res.

[10] Gupta E, Ballani N, Kumar M, Sarin SK (2015). Role of non-hepatotropic viruses in acute sporadic viral hepatitis and acute-on-chronic liver failure in adults. Indian J Gastroenterol.

[11] Rigopoulou EI, Smyk DS, Matthews CE, Billinis C, Burroughs AK, Lenzi M, Bogdanos DP (2012). Epstein-barr virus as a trigger of autoimmune liver diseases. Adv Virol.

[12] Vine LJ, Shepherd K, Hunter JG, Madden R, Thornton C, Ellis V, Bendall RP (2012). Characteristics of Epstein-Barr virus hepatitis among patients with jaundice or acute hepatitis. Aliment Pharmacol Ther.

[13] Ulug M, Celen MK, Ayaz C, Geyik MF, Hosoglu S (2010). Acute hepatitis: a rare complication of Epstein-Barr virus (EBV) infection. J Infect Dev Ctries.

[14] Gupta E, Bhatia V, Choudhary A, Rastogi A, Gupta NL (2013). Epstein-Barr virus associated acute hepatitis with cross-reacting antibodies to other herpes viruses in immunocompetent patients: report of two cases. J Med Virol.

[15] Sipeki N, Antal-Szalmas P, Lakatos PL, Papp M (2014). Immune dysfunction in cirrhosis. World J Gastroenterol.

[16] D'Amico G, Garcia-Tsao G, Pagliaro L (2006). Natural history and prognostic indicators of survival in cirrhosis: a systematic review of 118 studies. J Hepatol.

[17] Tandon P, Garcia-Tsao G (2011). Renal dysfunction is the most important independent predictor of mortality in cirrhotic patients with spontaneous bacterial peritonitis. Clin Gastroenterol Hepatol.

[18] Riggio O, Ridola L, Pasquale C, Nardelli S, Pentassuglio I, Moscucci F, Merli M (2011). Evidence of persistent cognitive impairment after resolution of overt hepatic encephalopathy. Clin Gastroenterol Hepatol.

[19] de Franchis R, Baveno VF (2010). Revising consensus in portal hypertension: report of the Baveno V consensus workshop on methodology of diagnosis and therapy in portal hypertension. J Hepatol.

[20] Bruix J, Sherman M (2011). American Association for the Study of liver D. management of hepatocellular carcinoma: an update. Hepatology..

[21] Salerno F, Gerbes A, Gines P, Wong F, Arroyo V (2007). Diagnosis, prevention and treatment of hepatorenal syndrome in cirrhosis. Gut..

[22] Gentilini P, Laffi G, La Villa G, Romanelli RG, Buzzelli G, Casini-Raggi V, Melani L (1997). Long course and prognostic factors of virus-induced cirrhosis of the liver. Am J Gastroenterol.

[23] Pugh RN, Murray-Lyon IM, Dawson JL, Pietroni MC, Williams R (1973). Transection of the oesophagus for bleeding oesophageal varices. Br J Surg.

[24] Kamath PS, Wiesner RH, Malinchoc M, Kremers W, Therneau TM, Kosberg CL, D'Amico G (2001). A model to predict survival in patients with end-stage liver disease. Hepatology..

[25] John JA, de Mattos AA, da Silva Miozzo SA, Comerlato PH, Porto M, Contiero P, da Silva RR (2015). Survival and risk factors related to death in outpatients with cirrhosis treated in a clinic in southern Brazil. Eur J Gastroenterol Hepatol.

[26] Merli M, Lucidi C, Giannelli V, Giusto M, Riggio O, Falcone M, Ridola L (2010). Cirrhotic patients are at risk for health care-associated bacterial infections. Clin Gastroenterol Hepatol.

[27] Bajaj JS, O'Leary JG, Reddy KR, Wong F, Olson JC, Subramanian RM, Brown G (2012). Second infections independently increase mortality in hospitalized patients with cirrhosis: the north American consortium for the study of end-stage liver disease (NACSELD) experience. Hepatology..

[28] Shi Y, Yang Y, Hu YR, Wu W, Yang Q, Zheng M, Zhang S (2015). Acute-on-chronic liver failure precipitated by hepatic injury is distinct from that precipitated by extrahepatic insults. Hepatology..

[29] De Pauw B, Walsh TJ, Donnelly JP, Stevens DA, Edwards JE, Calandra T, Pappas PG (2008). Revised definitions of invasive fungal disease from the European Organization for Research and Treatment of Cancer/invasive fungal infections cooperative group and the National Institute of Allergy and Infectious Diseases mycoses study group (EORTC/MSG) consensus group. Clin Infect Dis.

[30] Hu J, Zhao H, Lou D, Gao H, Yang M, Zhang X, Jia H (2018). Human cytomegalovirus and Epstein-Barr virus infections, risk factors, and their influence on the liver function of patients with acute-on-chronic liver failure. BMC Infect Dis.

[31] Moreau R, Jalan R, Gines P, Pavesi M, Angeli P, Cordoba J, Durand F (2013). Acute-on-chronic liver failure is a distinct syndrome that develops in patients with acute decompensation of cirrhosis. Gastroenterology..

[32] Jalan R, Saliba F, Pavesi M, Amoros A, Moreau R, Gines P, Levesque E (2014). Development and validation of a prognostic score to predict mortality in patients with acute-on-chronic liver failure. J Hepatol.

[33] Balfour HH, Odumade OA, Schmeling DO, Mullan BD, Ed JA, Knight JA, Vezina HE (2013). Behavioral, virologic, and immunologic factors associated with acquisition and severity of primary Epstein-Barr virus infection in university students. J Infect Dis.

[34] Balfour HH, Sifakis F, Sliman JA, Knight JA, Schmeling DO, Thomas W (2013). Age-specific prevalence of Epstein-Barr virus infection among individuals aged 6-19 years in the United States and factors affecting its acquisition. J Infect Dis.

[35] Petrova M, Muhtarova M, Nikolova M, Magaev S, Taskov H, Nikolovska D, Krastev Z (2006). Chronic Epstein-Barr virus-related hepatitis in immunocompetent patients. World J Gastroenterol.

